# Flavone acetic acid induces a G2/M cell cycle arrest in mammary carcinoma cells

**DOI:** 10.1038/sj.bjc.6690619

**Published:** 1999-08

**Authors:** N J Panaro, N C Popescu, S R Harris, U P Thorgeirsson

**Affiliations:** Laboratory of Cellular Carcinogenesis and Tumor Promotion, Laboratory of Experimental Carcinogenesis, Division of Basic Sciences, National Cancer Institute, NIH, Bethesda, MD 20892, USA

**Keywords:** FAA, cell cycle, mammary, rat

## Abstract

Flavone acetic acid (FAA) is a synthetic flavonoid that demonstrated extraordinary anti-tumour properties in murine models but was not effective in clinical trials. In an effort to better understand the molecular mechanisms by which FAA asserts its tumouricidal activities, we have examined the effect of FAA on the cell cycle. We observed FAA-mediated G2/M cell cycle arrest in mammary carcinoma cells at a concentration previously demonstrated to have anti-tumour effects in rodent models. The cell cycle arrest was accompanied by an increase in the P34^cdc2^ (cdc2) cyclin-dependent kinase activity. Morphological cytogenetic analysis demonstrated a colcemid-like effect of FAA on cytokinesis by causing accumulation of condensed C-metaphases of a sustained mitotic block. The cell cycle effect was blocked by the antioxidants ADPC and ascorbate, the superoxide scavenger Tiron, and the sphingosine kinase inhibitor L-cycloserine, but not by inhibitors of nitric oxide synthase. Based on these data, we propose that FAA may induce cell cycle arrest by stimulating the activity of acidic sphingomyelinase leading to the generation of reactive oxygen species. © 1999 Cancer Research Campaign


British Journal of Cancer (1999) 80(12), 1905–1911
© 1999 Cancer Research Campaign
Article no. bjoc.1999.0619

### Flavone acetic acid induces a G2/M cell cycle arrest in mammary carcinoma cells

**NJ Panaro[1], NC Popescu[2], SR Harris[1] and UP Thorgeirsson[1]**

[1]Laboratory of Cellular Carcinogenesis and Tumor Promotion, [2]Laboratory of Experimental Carcinogenesis, Division of Basic Sciences, National Cancer Institute, NIH, Bethesda, MD 20892, USA

**Summary** Flavone acetic acid (FAA) is a synthetic flavonoid that demonstrated extraordinary anti-tumour properties in murine models but was not effective in clinical trials. In an effort to better understand the molecular mechanisms by which FAA asserts its tumouricidal activities, we have examined the effect of FAA on the cell cycle. We observed FAA-mediated G2/M cell cycle arrest in mammary carcinoma cells at a concentration previously demonstrated to have anti-tumour effects in rodent models. The cell cycle arrest was accompanied by an increase in the $P34^{cdc2}$ (cdc2) cyclin-dependent kinase activity. Morphological cytogenetic analysis demonstrated a colcemid-like effect of FAA on cytokinesis by causing accumulation of condensed C-metaphases of a sustained mitotic block. The cell cycle effect was blocked by the antioxidants ADPC and ascorbate, the superoxide scavenger Tiron, and the sphingosine kinase inhibitor L-cycloserine, but not by inhibitors of nitric oxide synthase. Based on these data, we propose that FAA may induce cell cycle arrest by stimulating the activity of acidic sphingomyelinase leading to the generation of reactive oxygen species.

**Keywords**: FAA; cell cycle; mammary; rat

Flavone acetic acid (FAA, NSC 347512) is a synthetic flavonoid that possesses striking anti-tumour activity in a wide variety of solid tumours in murine models of cancer (Finlay et al, 1988; Cummings and Smyth, 1989; Hill et al, 1991; Bowler and Pearson, 1992). Experimental evidence suggests that FAA kills tumour cells indirectly by reducing tumour blood flow causing ischaemic conditions within the tumour (Bibby et al, 1989; Zwi et al, 1989; Madhevan and Hart, 1991) without effecting systemic blood flow (Bibby and Double, 1993). In addition to vascular collapse, the release of cytokines such as tumour necrosis factor (TNF)-α, interferon-α and interferon-γ by immune system cells plays an important role in the tumouricidal properties of FAA (Ching and Bagueley, 1987; Pratesi et al, 1990; Futami et al, 1991; Chabot et al, 1993). Furthermore, ample data exist suggesting that nitric oxide (NO) production within the tumour may also contribute to the cytotoxic activity of FAA either by direct toxicity to tumour cells or by altering tumour blood flow (Thomsen et al, 1990, 1991, 1992; Harris and Thorgeirsson, 1997). Unfortunately, FAA failed to illicit a similar response in human clinical trials, leading to a decreased interest in examining the molecular pathways involved in its anti-tumour properties in the mouse. A greater understanding of the molecular pathways involved in the anti-tumour actions of FAA might suggest rational structural modifications to FAA, combination strategies, and/or identify novel molecular targets for anti-tumour therapies.

In this study, we have chosen to examine the anti-proliferative effects of FAA. While the effects of other flavonoids on the cell cycle progression have been examined to extent, surprisingly no similar study has been performed with FAA. The flavonol quercetin arrests human gastric cancer cells at the G1/S boundary (Yoshida et al, 1990) whereas the isoflavone genistein blocks proliferation of the same cell line in G2/M (Matsukawa et al, 1993). The flavone apigenin has been shown to arrest mouse keratinocytes in G2/M (Sato et al, 1994; Lepley et al, 1996), while the flavone flavopirodol is capable of blocking cell proliferation in both G1 and G2 in A549 lung carcinoma cells (Bible and Kaufman, 1996; Carlson et al, 1996). Hence, there is no clear link between flavonoid structure and their ability to arrest cell growth in a particular phase of the cell cycle. We have found that FAA causes rat mammary carcinoma cells (and other cell types) to arrest in G2/M and that this effect is accompanied by increased cdc2 kinase activity.

*Received 27 August 1998*
*Accepted 4 March 1999*

*Correspondence to:* UP Thorgeirsson

### MATERIALS AND METHODS

### Cell culture

NMU cells (ATCC, Rockville, MD, USA) were obtained at passage number 21. Cells were maintained in Dulbecco's modified Eagle's medium (DMEM) containing 4 mM glutamine and 4.5 g glucose $l^{-1}$, and supplemented with 10% fetal bovine serum, 100 units penicillin $ml^{-1}$, 100 μg streptomycin $ml^{-1}$ and 25 mM HEPES buffer (Biofluids, Rockville, MD, USA). All experiments were conducted using cells between passages 25 and 48.

For all experiments, confluent flasks of NMU cells were trypsinized and split 1:10, 2–3 days prior to FAA treatment. Floating and adherent cells for flow cytometry were pelleted by centrifugation at 600 ***g*** for 5 min. For experiments involving the MTS/PMS assay, 2000 cells were seeded per well in replicates of six per experimental treatment in 96-well plates.

### Chemicals

Flavone 8-acetic acid (FAA) was obtained from the NIH Drug Synthesis Branch. Ammoniumpyrrolidinedithiocarbamate (ADPC) was obtained from Alexis Corporation (San Diego, CA, USA). *S*-ethylisothiourea-HBr (SEIU) and L-cycloserine were obtained from Biomol Research Laboratories (Plymouth Meeting, PA, USA). Radiolabelled [$\gamma$-$^{32}$P]ATP was obtained from Amersham Life Science, Inc. (Arlington, IL, USA). All other chemicals were obtained from Sigma Chemical Co. (St Louis, MO, USA).

### Flow cytometry

Cell pellets were resuspended in 0.5 ml of HBSS and then fixed by adding 4.5 ml of ice-cold 70% ethanol. Cells were stained with propidium iodide for cell cycle analysis using the Becton Dickinson Cycle Test Plus DNA Reagent Kit (Becton Dickinson, San Jose, CA, USA) and filtered through a 48 mM nylon mesh (Tetko, Briarcliff Manor, NY, USA). Samples of 25 000 events were collected on a Becton Dickinson FACSort (Becton Dickinson) using the Cell Quest software package (Version # 1.0) and gated to remove doublets and debris.

### Cell proliferation assay

Cell proliferation was quantitated using the MTS (3-(4,5-di-me-thylthiazol-2-yl)-5-(3-carboxymethoxyphenyl)-2-(4-sul-phonyl-2-H-tetrazolium, inner salt)–PMS (phenazine methosul-phate) method (Promega, Madison, WI, USA). Briefly, 20 µl of an MTS–PMS mixture (20:1) was added to 96-well plates containing 100 µl of culture media per well. Plates were incubated at 37°C for 1 h and absorbance read at 490 nm. Each well was run in replicates of six wells, with the high and low absorbances rejected and averages of the remaining four wells used to calculate cell proliferation.

### Immunoblot analysis

Cell pellets were resuspended in 50 µl of loading buffer (Novex, San Diego, CA, USA), sonicated (2 × 10-second pulses), vortexed briefly, snap-frozen in liquid nitrogen and stored at –80°C until assayed. Protein concentrations were quantitated using the BCA method (Pierce, Rockford, IL, USA) and 20-µg aliquots of total protein were loaded in each lane, electrophoresed and transferred to a nitrocellulose membrane. The membrane was placed in 5% milk (BioRad) in TBS (Tris-buffered saline) for 8 h, rinsed three times in TTBS (0.05% Tween-20 in TBS), followed by 16-h incubation in SuperBlock (Pierce). Incubation with 1:1000 dilution of rabbit anti-rat cyclin B1 (Santa Cruz, Santa Cruz, CA, USA) was carried out for 8 h, washed three times with TTBS, incubated in biotinylated goat anti-rabbit antibody (1:2000 in TTBS) for 1 h, washed three times with TTBS, incubated with strepavidin–biotinylated alkaline phosphatase complex (1:3000 in TTBS) for 1 h, and washed again three times with TTBS. Bands were detected using Bio-Rad AP Color Development kit.

### Mitotic index and cytogenetic analysis

Exponentially growing NMU cells were treated with FAA (250 µg ml$^{-1}$) or colcemid (0.5 µg ml$^{-1}$). This concentration of FAA was chosen because it was shown by flow cytometry to be the most effective in causing a sustained G2/M block. Mitotic indices and chromosome ploidy were determined after 8, 16, 24, 36 and 48 h of FAA or colcemid treatment. For the mitotic indices the cells were cultured on glass coverslips. At the indicated time points, the medium was removed, the cells were washed in PBS, fixed in absolute methanol, stained with DAPI or Giemsa, and mounted. Two coverslips (1000 cells per coverslip) were examined for each time point. Chromosome ploidy was determined in exponentially growing cells, cultured in 100-mm petri dishes. The chromosomes were prepared by a standard technique, using hypotonic potassium chloride treatment, acetic acid–methanol fixation, and air-drying of the slides. For each time point, 200 Giemsa-stained metaphases with minimal chromosome overlapping were recorded and the chromosomes were counted on the screen.

### RNA blotting

Total cellular RNA was obtained from the cell pellets using Qiagen Rneasy Kit (Qiagen, Chatsworth, CA, USA), quantitated using the GENE Quant II (Pharmacia, Piscataway, NJ, USA) and concentrated by use of a SpeedVac. Twenty micrograms of total RNA were denatured in formaldehyde and formamide and electrophoresed through a 1% agarose gel containing formaldehyde alongside the appropriate RNA molecular weight ladder (Gibco-BRL, Gaithersburg, MD, USA). Ethidium bromide-stained gels were photographed to confirm RNA quality and equal loading of lanes. Gels were washed four times in DEPC water and transferred to a nylon-supported nitrocellulose membrane. The membrane was pre-hybridized with ExpressHyb (Clontech, Palo Alto, CA, USA) for 60 min at 68°C, followed by hybridization with a cyclin B1 probe made by random primer labelling of a 400 bp rat cyclin B1 cDNA (kind gift of Dr Michael Jensen, NCI) for 60 min at 68°C, washed in 2 × sodium–saline citrate (SSC)/0.05% sodium dodecyl sulphate (SDS) (3 × 10 min, room temperature), 0.1 × SSC/0.1% SDS (10 min, 50°C), and 0.1 × SSC/0.1 % SDS (30 min, 50°C). Membranes were exposed to a phosphoimaging screen for 48–72 h and images captured and band intensities quantified using a Storm phosphoimager. Dehybridization of probes from the membranes was achieved by washing in 0.5% SDS (10 min, 90°C). Membranes were then re-hybridized and intensities were quantitated using a glyceraldehyde 3-phosphate dehydrogenase (GAPDH) cDNA probe (kind gift of Dr E Fernandez-Salas and Dr S Yuspa, NCI) as described above.

### Cdc2 kinase activity assay

Cell pellets were obtained as described above. P34$^{cdc2}$ kinase activity was determined using the SignaTECT cdc2 Protein Kinase Assay System (Promega). Each sample was assayed in triplicate (5 µl per replicate) as described by Promega. Protein concentrations were determined using the Pierce BCA method.

### RESULTS

### FAA induces a G2/M cell cycle arrest

Figure 1 shows the effect of FAA on the cell cycle of the NMU rat mammary carcinoma cells as a function of concentration and time. A sustained G2/M cell cycle arrest was observed at 250 µg ml$^{-1}$ of FAA over a 24-h time period while no effect was observed at

© 1999 Cancer Research Campaign

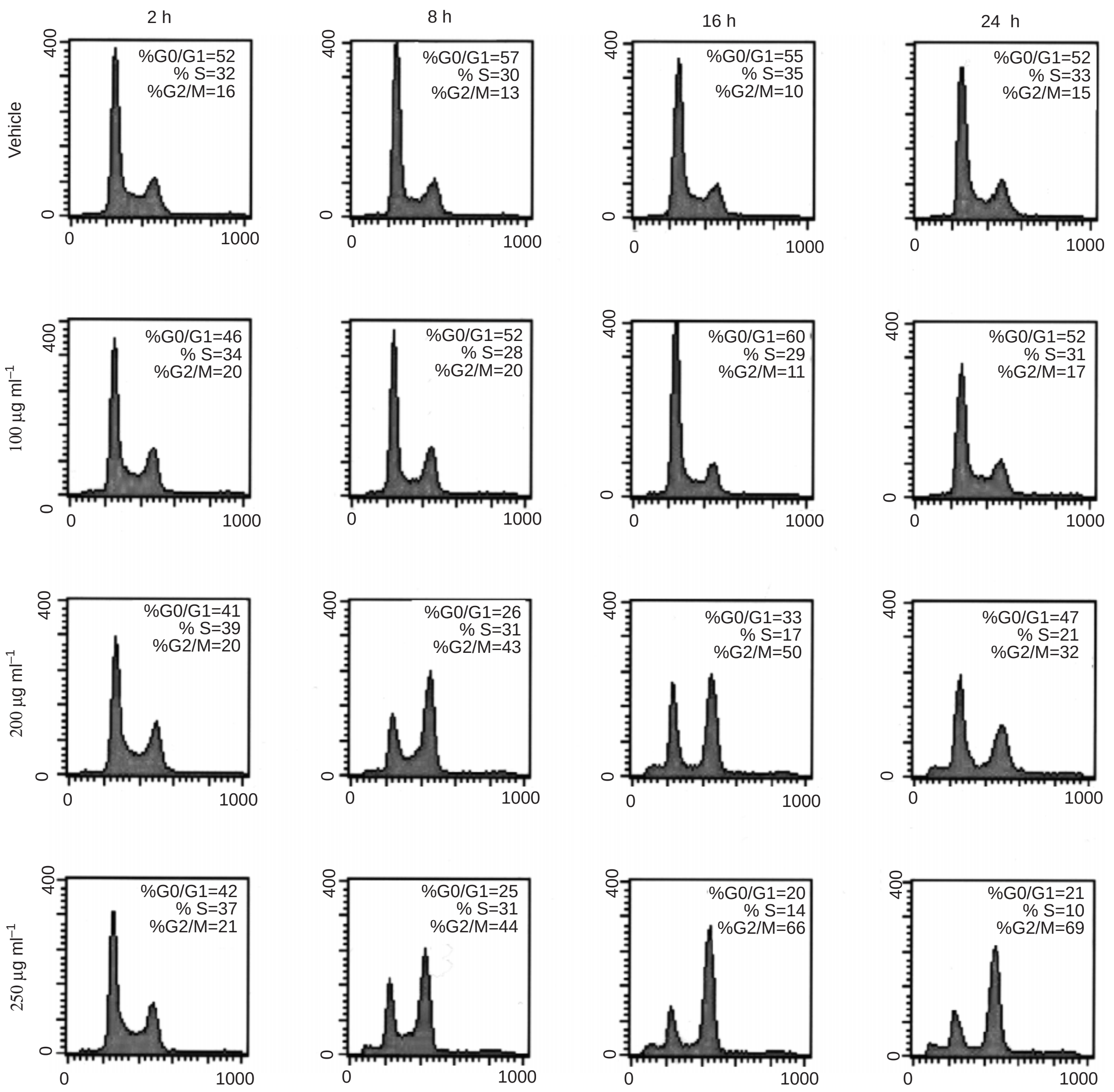

**Figure 1** FAA-mediated cell cycle effects on rat mammary carcinoma cells, analysed by flow cytometry

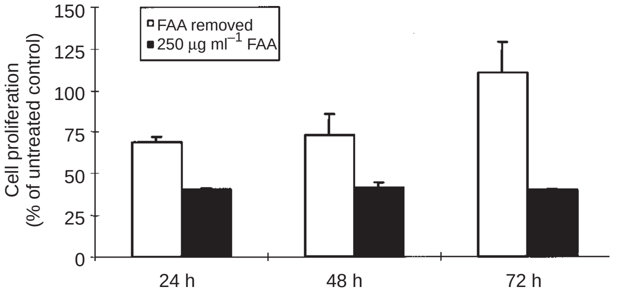

**Figure 2** Recovery of rat mammary carcinoma cell proliferation after removal of FAA. At 72 h after removal of FAA, cellular proliferation was statistically identical to that of the untreated control ($P < 0.05$, $n = 3$). Error bars represent standard error of the means

100 µg ml$^{-1}$. Interestingly, 200 µg ml$^{-1}$ of FAA caused a transient G2/M cell cycle arrest which was apparent at 8 h but had reversed towards the control level at 24 h. To determine if the cell cycle arrest was reversible after the NMU cells were exposed to 250 µg ml$^{-1}$ FAA for 24 h, cell proliferation was measured after the removal of the FAA, using the MTS–PMS method. Figure 2 shows that cell proliferation recovered to levels of the untreated control 72 h after the removal of FAA.

### Cyclin B1 levels in NMU cells exposed to FAA

Northern blot analysis showed 1.6 and 2.4 kb transcripts for cyclin B1. Densitometric readings showed no changes in the cyclin B1 RNA levels (Figure 3A) and immunoblot analysis showed no differences following 24-h exposure to FAA (250 µg ml$^{-1}$) (Figure 3B).

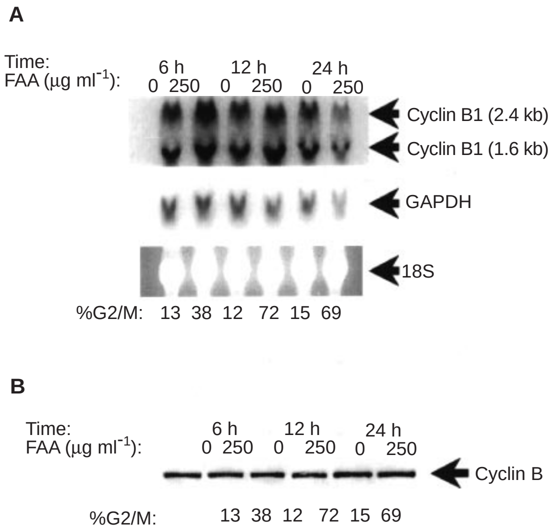

**Figure 3** Effects of FAA on cyclin B1 levels. (**A**) Northern blot analysis of cyclin B1 RNA expression. Cyclin B1 expression is represented by 1.6- and 2.4-kDa transcripts. To evaluate the quantity and quality of the RNA, the blots were rehybridized with a GADPH probe and stained with ethidium bromide. (**B**) Immunoblot analysis for cyclin B1. The percentage of cells in the G2/M phase are shown below the blots

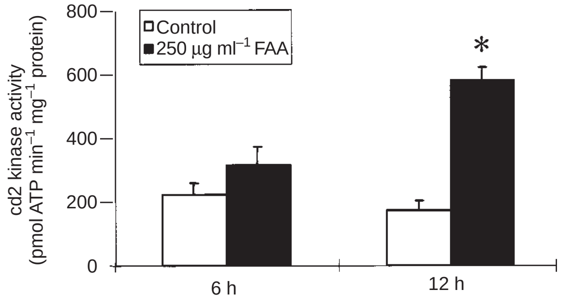

**Figure 4** Cdc2 kinase activity in FAA-treated NMU cells. Cdc2 kinase activity was significantly increased after a 12-h exposure to 250 μg ml$^{-1}$ FAA ($P < 0.05$, $n = 3$). Error bars represent standard error of the means

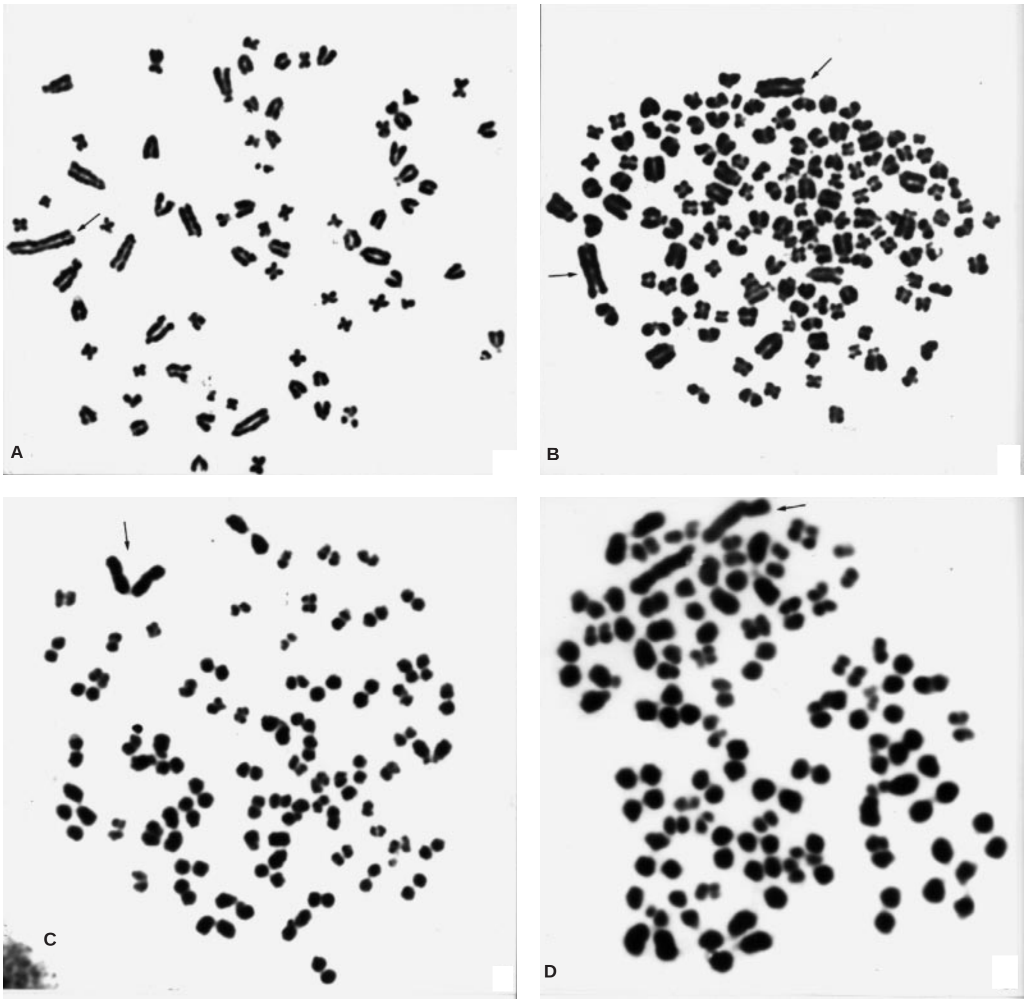

**Figure 5** Representative metaphases from the FAA-treated NMU rat mammary carcinoma cells: (**A**) triploid metaphases; (**B**) hexaploid metaphases; (**C**) C-mitosis with centromeres still attached; (**D**) C-mitosis with centromeres completely separated. Arrows indicate large acrocentric chromosomes which were characteristic for the NMU rat mammary carcinoma cells

 *© 1999 Cancer Research Campaign*

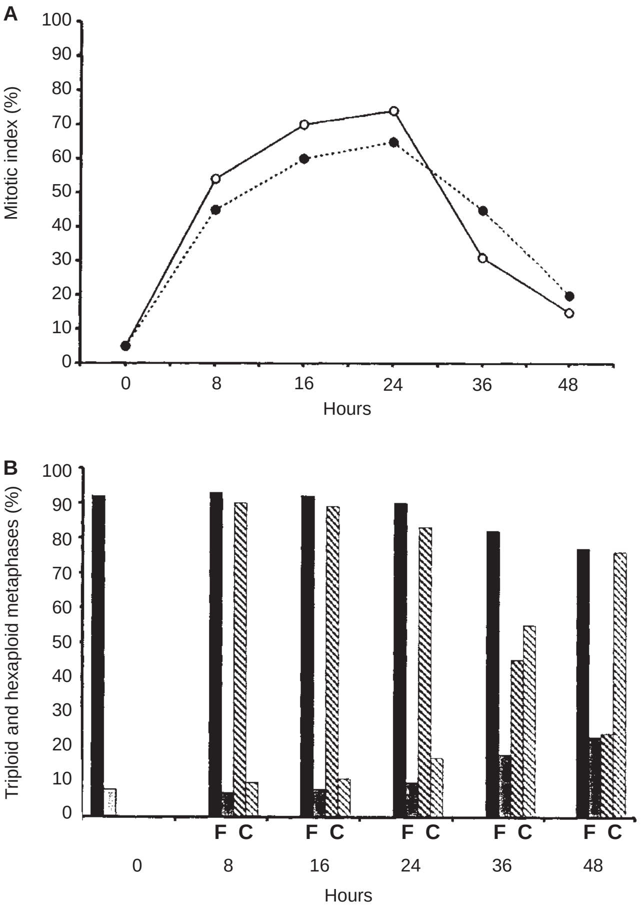

**Figure 6** Mitotic indices and ploidy in the NMU rat mammary carcinoma cells, treated with FAA or colcemid for 8, 16, 24, 36 and 48 h. (**A**) Mitotic indices at indicated time points of FAA (dotted line) and colcemid (solid line) treatment. (**B**) Chromosome ploidy was determined in FAA (F) and colcemid (C) treated cells at indicated time points as per cent (%) triploid (3n) and hexaploid (6n) metaphases. The columns representing metaphases from the FAA-treated cells are: triploid (solid black); hexaploid (heavy stripes). The columns representing metaphases from the colcemid treated cells are (heavy stripes); hexaploid (light stripes)

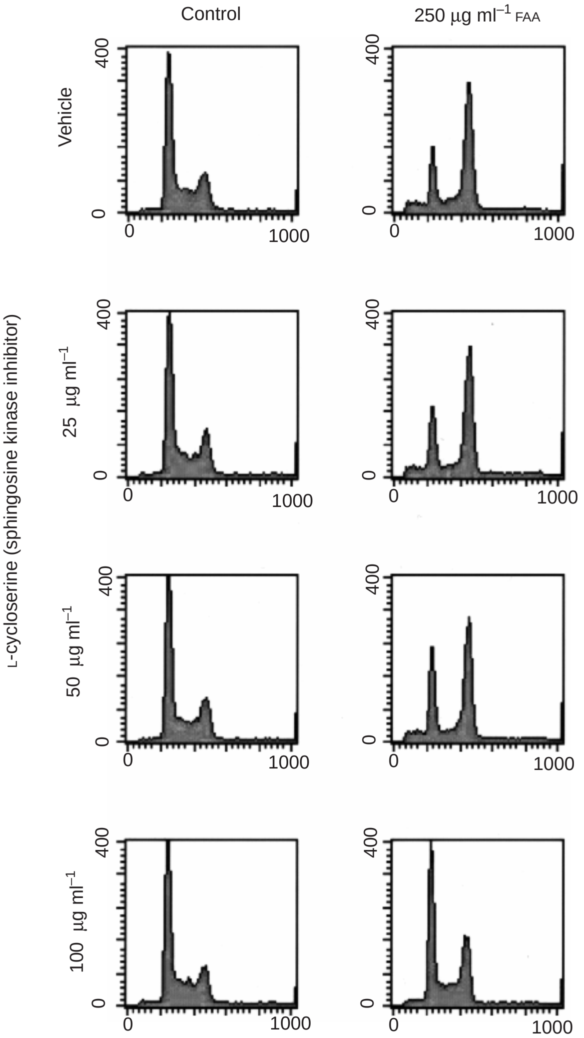

**Figure 7** Inhibitory effects of the sphingosine kinase inhibitor, L-cycloserine, on the FAA-mediated cell cycle effect, analysed by flow cytometry

### Cdc2 kinase activity is increased by FAA

Figure 4 shows cdc2 kinase activity in control and FAA-treated NMU cells 6 and 12 h after 250 µg $ml^{-1}$ FAA treatment. Insignificant increase in the kinase activity was observed at 6 h, whereas a statistically significant threefold increase was observed after a 12-h exposure to 250 µg $ml^{-1}$ FAA.

### Cytogenetic analysis

The NMU rat mammary carcinoma cell line displayed an aneuploid chromosome constitution with an abnormal chromosome number and structural abnormalities (Figures 5A,B and 6). A predominant population (92% of the cells) had a triploid number, ranging from 65 to 74 chromosomes (3n). A minor cell population had near hexaploid number, ranging from 130 to 146 (6n). The triploid cells had a distinctive large abnormal acrocentric chromosome which was duplicated in the hexaploid cells (Figure 5A,B). Morphological analysis was carried out on the FAA-G2/M blocked cells and compared with cells treated with colcemid, an effective mitotic arrestant. There were minor differences in the mitotic indices between the FAA and colcemid treated cells with a peak mitotic accumulation for both compounds at the 24-h time point (Figure 6A).

To determine the nature of the mitotic block, chromosomes were examined after 8, 16, 24, 36 and 48 h of FAA or colcemid treatment. The two compounds showed a similar distribution of triploid and hexaploid cells up to 16 h (Figure 6B). Thereafter, the incidence of hexaploidy increased significantly in the colcemid treated cells and became the predominant population at 48 h. In the FAA treated cells, however, only a small increase in the incidence of hexaploid cells occurred after the 16th time point (Figure 6B). This suggests that only a small fraction of the cells at higher DNA ploidy, which are reflected in the G2/M peak of the histograms (Figure 1), are G1 cells. When FAA was removed after 6 h exposure, there was no increase in hexaploid cells for a period up to 48 h (data not shown).

Metaphases with diplochromosomes, reflecting endoreduplication, were observed in 2–3% of the colcemid-treated cells, but in none of the FAA-treated cells (Figure 6). A striking feature of the cells arrested in metaphase after FAA treatment was the appearance of C-mitosis, known to be caused by colcemid and named accordingly (Therman, 1980). C-metaphases exhibited separated

© 1999 Cancer Research Campaign chromatids with the centromere still attached in most mitotic figures and complete separation in some metaphases (Figure 5C, D). The incidence of C-metaphases increased from 20% at 8 h to 40% and 70% at 16 h and 24 h of FAA treatment. A similar frequency of C-mitosis was observed in the colcemid treated cultures. C-mitosis was not observed in cultures exposed to either chemical for 3–4 h prior to cell harvest.

### L-cycloserine, an inhibitor of sphingosine biosynthesis, blocks the ability of FAA to arrest cell proliferation

Co-administration of L-cycloserine with FAA blocked the cell cycle arrest in a dose-dependent manner (Figure 7). At 25 $\mu g\ ml^{-1}$, L-cycloserine had little effect on the FAA-induced cell cycle arrest. However, at 100 $\mu g\ ml^{-1}$ L-cycloserine blocked the FAA-induced G2/M cell cycle arrest.

### DISCUSSION

Previous research conducted in our laboratory has demonstrated the ability of FAA to inhibit the proliferation of human umbilical vein endothelial cells (HUVEC) and human lung microvascular endothelial cells (HMVEC-L) in vitro (Lindsay et al, 1996). While the present study has mainly focused on mammary carcinoma cells, we observed a G2/M cell cycle arrest in other cell types tested, including HUVEC and HMVEC-L, as well as rat liver epithelial cells (RLE) and v-*raf*/v-*myc* transformed RLE (unpublished observations). The G2/M arrest in mammary carcinoma cells in vitro occurred at doses previously shown to have anti-tumour effects in rodents.

Major cell cycle transitions are regulated by a family of serine/threonine kinases known as cyclin-dependent kinases. cdc2 regulates the G2/M transition by phosphorylating a key group of proteins (Mitra et al, 1996; Wu et al, 1996). Protein levels of cdc2 remain constant throughout the cell cycle and its kinase activity is therefore not regulated at the level of transcription but rather by its association with cyclin B and by its phosphorylation state (Draetta and Beach, 1988). Formation of cdc2–cyclin B complex is necessary for the G2/M transition to take place and the cells to enter mitosis. The FAA-mediated cell cycle arrest was not associated with decreased cyclin B1 levels. Thus it would appear that the observed G2/M cell cycle arrest was not the result of decreased cyclin B1 levels. Suppressed cyclin B levels have been observed in the G2/M arrest of mouse keratinocytes treated with the flavonoid apigenin (Lepley et al, 1996) and the G2/M arrest in HeLa cells following ionizing radiation (Muschel et al, 1991, 1993).

Interestingly, there was an increase in cdc2 kinase activity after a 12-h exposure to FAA. Elevated levels of cdc2 kinase activity have been observed in HeLa cells treated with colcemid, nocodozole or taxol, each of which causes cells to arrest in G2/M, and that this cdc2 hyperactivation may be necessary for taxol-induced apoptosis (Kung et al, 1990; Donaldson et al, 1994).

Cytogenetic analysis of the NMU rat mammary carcinoma cells showed that FAA had colcemid-like effect with accumulation of C-metaphases. A recent report on mutations of mitotic checkpoint genes in human colorectal cancer cell lines has provided a new insight into the understanding of chromosomal instability which leads to aneuploidy in cancer (Cahill et al, 1998). Colorectal cancer cell lines with chromosomal instability were found to be unresponsive to a mitotic block by colcemid due to a mutational inactivation of the human homologue of the yeast BUB1 gene (Cahill et al, 1998). In our study, the aneuploid NMU rat mammary carcinoma cells showed a normal response to colcemid and were also arrested by FAA. Further characterization of the cell cycle effect of FAA in near diploid versus aneuploid cancer cell lines may reveal important new findings on the cell cycle effect of FAA, which could resurrect an interest in this previously failed chemotherapeutic agent.

It has been proposed that FAA may act as a free radical and possess pro-oxidant properties (Candeias et al, 1993; Cao et al, 1997; Hodnick et al, 1997). This is supported by our findings showing that the antioxidants ADPC and the superoxide scavenger Tiron block the FAA-mediated cell cycle arrest (data not shown). We also considered the possibility that FAA as a free radical and a stimulator of pro-inflammatory cytokines could act through stimulation of acidic pH-dependent sphingomyelinase (ASMase) (Feinstein et al, 1995; Adam et al, 1996). L-cycloserine, an ASMase inhibitor, was found to block the FAA-mediated cell cycle effect. Studies by Pahan et al (1998) have shown that simultaneous treatment of astrocytes with ASMase and cytokines resulted in increased levels of NO. Hence, we asked the question if ASMase was involved in the cell cycle arrest through stimulation of the NO pathways. It was particularly relevant since our previous study on FAA-mediated anti-tumour effect on the mouse tumours implicated involvement of NO (Harris et al, 1997). However, NOS inhibitors had no effect on the FAA-mediated cell cycle arrest in the present study (not shown), suggesting the NO was not involved.

We have demonstrated that FAA induces a sustained G2/M arrest comparable to that caused by colcemid in rat mammary carcinoma cells. The data indicate that cdc2 is targeted by the growth arresting effect of FAA and that generation of reactive oxygen species may play a role.

### REFERENCES

Bibby MC and Double JA (1993) Flavone acetic acid: from laboratory to clinic and back. *Anti-Cancer Drugs* **4**: 3–13

Bibby MC, Double JA, Loadman PM and Duke CV (1989) Reduction of tumor blood flow by flavone acetic acid: a possible component of therapy. *J Natl Cancer Inst* **81**: 216–220

Bible KC and Kaufman SH (1996) Flavopiridol: a cytotoxic flavone that induces cell death in noncycling A549 human lung carcinoma cells. *Cancer Res* **56**: 4856–4861

Bowler K and Pearson JA (1992) Long term effects of flavone acetic acid on the growth of a rat tumor. *Anticancer Res* **12**: 1275–1280

Cahill DP, Lenguaer C, Yu J, Riggins GJ, Wilson KV, Markowitz SD, Kinzler KW, and Vogelstein B (1998) Mutations of mitotic checkpoint genes in human cancers. *Nature* **392**: 300–303

Candeias LP, Everett SA and Wardman P (1993) Free radical intermediates in the oxidation of flavone-8-acetic acid: possible involvement in its antitumor activity. *Free Radical Biol Med* **15**: 385–394

Cao G, Sofic E and Prior RL (1997) Antioxidant and prooxidant behavior of flavonoids: structure–activity relationships. *Free Radical Biol Med* **22**: 749–760

Carlson BA, DuBay MM, Sausville EA, Brizuela L and Worland PJ (1996) Flavopiridol induces G1 arrest with inhibition of cyclin-dependent kinase (CDK)2 and CDK4 in human breast carcinoma cells. *Cancer Res* **56**: 2973–2978

Chabot GG, Branellec D, Sassi A, Armand JP, Gouyette A, Chouaib S (1993) Tumour necrosis factor-alpha plasma levels after flavone acetic acid administration in man and mouse. *Eur J Cancer* **29A(5)**: 729–733

Ching LM and Baguley BC (1987) Induction of natural killer cell activity by the antitumor compound flavone acetic acid (NSC 347512). *Eur J Cancer Clin Oncol* **23:** 1047–1050

*© 1999 Cancer Research Campaign*

Cummings J and Smyth JF (1989) Flavone 8-acetic acid: our current understanding of its mechanism of action in solid tumours. *Cancer Chemother Pharmacol* **24**: 269–272

Donaldson KL, Goolsby GL, Kiene PA and Wahl AF (1994) Activation of p34[cdc2] coincident with taxol-induced apoptosis. *Cell Growth Diff* **5**: 1041–1050

Draetta G and Beach D (1988) Activation of cdc2 kinase during mitosis in human cells: cell cycle-dependent phosphorylation and subunit rearrangement. *Cell* **54**: 17–26

Feinstein E, Kimchi A, Wallach D, Boldin M and Varfolomeev E (1995) The death domain: a module shared by proteins with diverse cellular functions. *Trends Biochem Sci* **20**: 342–344

Finlay GJ, Smith GP, Fray LM and Baguley BC (1988) Effect of flavone acetic acid on Lewis lung carcinoma: evidence for an indirect effect. *J Natl Cancer Inst* **80**: 241–245

Futami H, Eader LA, Komschlies KL, Bull R, Gruys ME, Ortaldo JR, Young HA, and Wiltrout RH (1991) Flavone acetic acid directly induces expression of cytokine genes in mouse splenic leukocytes but not in human peripheral blood leukocytes. *Cancer Res* **51**: 6596–6602

Harris SR and Thorgeirsson UP (1997) Flavone acetic acid stimulates nitric oxide and peroxynitrate production in subcutaneous mouse tumors. *Biochem Biophys Res Commun* **235**: 509–514

Hill SA, Williams KB and Denekemp J (1991) Studies with a panel of tumours having a variable sensitivity to FAA, to investigate its mechanism of action. *Int J Radiat Biol* **60**: 379–384

Hodnick WF, Duval DL and Pardini RS (1994) Inhibitor of mitochondrial respiration and cyanide-stimulated generation of reactive oxygen species by selected flavonoids. *Biochem Pharmacol* **47**: 573–580

Kung AL, Sherwood SW and Schimke RT (1990) Cell line-specific differences in the control cell cycle progression in the absence of mitosis. *Proc Natl Acad Sci USA* **87**: 9553–9557

Lepley DM, Li B, Birt DF and Pelling JC (1996) The chemopreventive flavonoid apigenyin induces G2/M arrest in keratinocytes. *Carcinogenesis* **17**: 2367–2375

Lindsay CK, Gomez DE and Thorgeirsson UP (1996) Effect of flavone acetic acid on endothelial cell proliferation: evidence for antiangiogenic properties. *Anticancer Res* **16**: 425–432

Madhevan V and Hart IR (1991) Divergent effects of flavone acetic acid on established versus developing tumour blood flow. *Br J Cancer* **63**: 889–892

Matsukawa Y, Marui N, Sakai T, Satomi Y, Yoshida M, Matsumoto K, Nishino H and Aoike A (1993) Genistein arrests cell cycle progression at G2-M. *Cancer Res* **53**: 1328–1331

Mitra J and Schultz RM (1996) Regulation of the acquisition of meiotic competence in the mouse: changes in the subcellular localization of cdc2, cyclin B1, cdc25 and weel, and in the concentration of these proteins and their transcripts. *J Cell Sci* **109**: 2407–2415

Muschel RJ, Zhang HB, Iliakis G and McKenna WG (1991) Cyclin B expression in HeLa cells during the G2 block induced by ionizing radiation. *Cancer Res* **51**: 5113–5117

Muschel RJ, Zhang HB and McKenna WG (1993) Differential effect of ionizing radiation on the expression of cyclin A and cyclin B in HeLa cells. *Cancer Res* **53**: 1128–1135

Pahan K, Sheikh FG, Khan M, Namboodiri AM and Singh I (1998) Sphingomyelinase and ceramide stimulate the expression of inducible nitric oxide synthase in rat primary astrocytes. *J Biol Chem* **273**: 2591–2600

Pratesi G, Rodolpho M, Rovetta G and Parminiani G (1990) Role of T cells and tumour necrosis factor in antitumor activity and toxicity of flavone acetic acid. *Eur J Cancer* **26**: 1079–1083

Sato F, Matsukawa Y, Matsumoto K, Nishino H and Sakai T (1994) Apigenin induces morphological differentiation and G2-M arrest in rat neuronal cells. *Biochem Biophys Res Commun* **204**: 578–584

Therman E (1980) *Human Chromosomes: Structure, Behavior, Effects*. Springer-Verlag: New York

Thomsen LL, Ching LM and Baguley BC (1990) Evidence for the production of nitric oxide by activated macrophages treated with the antitumor agents flavone-8-acetic and xanthenone-4-acetic acid. *Cancer Res* **50**: 6966–6970

Thomsen LL, Ching LM, Zhuang L, Gavin JB and Baguley BC (1991) Tumor-dependent increased plasma nitrate concentrations as an indication of the anti-tumor effect of flavone-8-acetic acid and analogues in mice. *Cancer Res* **51**: 77–81

Thomsen LL Ching LM, Joseph WR, Baguley BC and Gavin JB (1992) Nitric oxide production in endotoxin-resistant C3H/HeJ mice stimulated with flavone-8-acetic acid and xanthenone-4-acetic acid analogues. *Biochem Pharmacol* **43**: 2401–2406

Wu L, Shiozaki K, Aligue R and Russell P (1996) Spatial organization of the Nim1-Wee1-cdc2 mitotic control network in *Schizosaccharomyces pombe*. *Mol Biol Cell* **7**: 1749–1758

Yoshidia M, Sakai T, Hosokawa N, Marui N, Matsumoto K, Fujioka A, Nishino H and Aoike A (1990) The effect of quercetin on the cell cycle progression and growth of human gastric cancer cells. *FEBS Lett* **260**: 10–13

Zwi LJ, Baguley BC, Gavin JB and Wilson WR (1989) Blood flow failure as a major determinant in the antitumor action of flavone acetic acid. *J Natl Cancer Inst* **81**: 1005–1013

Zwi LJ, Baguley BC, Gavin JB and Wilson WR (1990) The use of vascularised spheroids to investigate the action of flavone acetic acid on tumor blood vessels. *Br J Cancer* **62**: 231–237

© 1999 Cancer Research Campaign 
